# An unusual presentation of an unusual disease: infective endocarditis: a case report and review of the literature

**DOI:** 10.1186/1757-1626-1-292

**Published:** 2008-10-31

**Authors:** Kishore Reddy Male, Antony Mathews, Johanna Mower

**Affiliations:** 1 University Hospital of Wales, Cardiff, CF14 4XW, UK

## Abstract

It is normal to have patients to present with agitation to the emergency department. The history could be suggestive of the diagnosis in most. When an apparently healthy young man comes to the hospital agitated, without any history of fever or trauma, it is not an easy one to diagnose. This is a case report of a 35 year old gentleman who presented with such a history and the final diagnosis turned out to be a usual disease. Hence any patient presenting with neurological manifestations as agitation, one should consider cardiac causes such as Infective Endocarditis if no other obvious cause is found initially.

## Case report

A 35 year old unemployed Caucasian gentleman was brought to the emergency unit of a University Hospital by ambulance in a agitated and combative state. He was an insulin dependent diabetic and his blood sugar was always brittle. He smoked 5–10 cigarettes a day and drank alcohol in moderate quantity. His mother was a tablet controlled diabetic with good glycemic control. It was impossible to do any observations or examine the patient. The family physician letter said patient is a diabetic on insulin and he had history of misuse of amphetamines. The patient was accompanied by his mother. Mother stated that the patient had flu like symptoms for a week and hence was staying with her.

On the day of presentation, he woke up from sleep feeling unwell and complained he had pain all over the body. He was taken to his family physician, who gave a referral letter to nearby hospital. On the way to the hospital in the car, he started becoming agitated and irritable. Mother felt he was going into hypoglycaemia. She called an ambulance. When the ambulance crew reached there, his BM was 1.5 mmol/L. They gave him 350 mls of 10% dextrose IV. His BM came to 20, but his clinical situation remained the same and later they brought him down to the emergency unit. Mother acknowledged that he does take recreational drugs. But she felt, in the preceding week, when he was with her it is very unlikely that he had been taking anything.

In the emergency unit his temp was 37.3c. His pupils were 4–5 mm in size reacting sluggishly to light, GCS was worked out to be 11 (M5, E4, V2). No other clinical examination was logistically possible.

Based on the history of intake of amphetamine, a working diagnosis of amphetamine overdose was made. Bloods samples were taken for all routine tests; toxicology screen and arterial blood gases were also performed. He was given diazemules IV in 2.5 mg alliquotes to a total of 10 mgs. A further dose of Haloperidol 5 mg IM was also given without much effect. More than an hour had passed by then. Since no further improvement of the clinical had occurred a decision was made to intubate him, do a CT scan and follow it up with lumbar puncture.

Just before the patient was about to be intubated, his temperature went up to 38.2 c and later to 39.6. On turning him, multiple purpuric non blanching rash could be seen on his back. Patient was intubated. Patient was given 2 gms of Ceftriaxone after taking blood for culture. CT scan done was negative. While in ICU further examination done revealed, systolic murmur at the apex. The next day patient had an Echocardiogram done which showed severe mitral regurgitation with vegetation and a mild tricuspid regurgitation.

A firm diagnosis of infective endocarditis was made and was treated for one.

Patient went on to have right frontal lobe infarct. He developed a septic left ankle for which ankle wash out was carried out. Patient had cardiac failure and cardiogenic shock following which he had mitral valve replacement with biologic prosthetic valve. He also developed a left foot drop which was attributed to pressure palsy or diabetic neuropathy. He had pneumonia with pleural effusion as well. A full six week course of intravenous antibiotics was completed. All this happened during this one time admission in the hospital. He recovered gradually and was then discharged home.

## Discussion

Confusion is a mental and behavioral state of reduced comprehension, coherence and capacity to reason. A state of confusion that is accompanied by agitation, hallucination, tremor and illusions is termed delirium. Is always a manifestation of a disorder of nervous system.

This could be a manifestation of a head injury, a seizure, drug toxicity, a metabolic disorder resulting from hepatic, renal, pulmonary or cardiac failure, systemic infection meningitis or encephalitis or a chronic dementing disease.

Even though rare, it is very clear that it could be a manifestation of Infective Endocarditis (IE) as well.

Infective endocarditis (IE) is an uncommon clinical entity that, if unrecognized, leads to serious morbidity and mortality [1] Early recognition of IE requires understanding of its epidemiology, risk factors, clinical presentations and physical examination signs Unrecognized and untreated, IE is invariably fatal. [1].

There is no dearth of literature mentioning the complexity and subtlety of Infective Endocarditis. In Sir William Oslers' words " Few disease present greater difficulties in the way of diagnosis than infective endocarditis, difficulties in many cases are practically insurmountable"

### Epidemiology

In developed countries the incidence is about 2.4 – 11.9 cases per 100,000 patients/yr. 50% of patients are between 31 and 60. The contributing factors are increased prevalence of degenerative heart disease, and increased use of invasive medical procedures. Men are more commonly affected than females. The majority of cases occur in those, with predisposing identifiable, cardiac structural abnormality. (Congenital or acquired), or has the recognized risk factors of the disease, such as injection drug use (IDU), indwelling catheters, poor dental hygiene or infection with human immunodeficiency virus (HIV). Incidence of IE is highest with native valve (NVE) which is 59–70%, IDU 11–16%, prosthetic valve IE (PVE) 14 – 30%. For NVE in the developing world, mitral valve prolapse is the most common, predisposing cardiac lesion for IE.

For IDUs the estimated risk is 2–5% per year. It generally is on the right side of the heart and tricuspid valve is affected in the vast majority of cases. Prosthetic valve IE (PVE) occurs in 3 to 6% of prosthetic valve recipients. The risk is highest in the first 6 months and then declines to 0.2–0.4% per year.

### Clinical Features

The commonest symptom of IE is fever.

A fever usually more than 38c is present. It may be absent in elderly patients, patients on antibiotics or antipyretics, those with congestive cardiac failure or renal failure. Other features are chills (40%), weakness (40%), dypnoea (40%), anorexia (25%), cough (25%), malaise (25%), skin lesions (20%), nausea/vomiting, headache (20%), stroke, chest pain, abdominal pain, back pain. Commonest sign is heart murmur (85%), petichae (20 – 40), splenomegaly (20 – 57), Embolic phenomenon > 50%.

A change in the mental status account for only 10–15% of the presenting symptoms. This is due to embolic stroke.

### Unusual presentations

Infective endocarditis is well known to present in many obscure ways. Non-specific clinical signs of IE are common, while highly specific signs are rare, and many of the symptoms commonly found in IE are ubiquitous in the population. For example, fatigue had an odds ratio for IE of ~3.5, but at any one time, 18.3% of the general population report substantial fatigue lasting > 6 months.

There is report of a patient who complained of arthralgias and arthritis for 1 month without fever. In the second month the patient developed asymmetrical polyarthritis with fever. It eventually turned out to be infective endocarditis. [2]

Another patient with IE presented with dysphagia and weight loss in an elderly patient (The patient's longstanding underlying bacteremia was thought to have caused pro-inflammatory changes leading to alterations in the neuronal environment affecting peripheral nerve function) [3]. Other unusual presentations include presentation with meningitis [4], presentation with testicular swelling, pneumonia and septicemia in a teenager[5]. Acute myocardial Infarction [6], with Sweet's syndrome [7]. (Febrile neutrophilic dermatosis – skin condition in some cancer patients)

In a study of patients with pyrexia of unknown origin (PUO), it was the third most common infectious disease (9.6%) [8]

### Neurological manifestation of IE

Mental state change occurs in 10 – 15% of patients with IE. Neurological manifestations accounts for about 20 – 40% in IE. Neurological presentation of IE are, strokes, TIA, purulent or aseptic meningitis, intracranial haemorrhage, headache seizure or encephalopathy.

In the majority of episodes with neurological manifestation, the neurological manifestation is evident before treatment is started, being the first sign of IE. Neurological complications are significantly associated with *Staphylococcus aureus *infection [9]

### Pathophysiology

Neurological complications of infective endocarditis (IE) occur as a result of embolization from endocardial vegetation. This results in the occlusion of cerebral arteries. An ischemic stroke or a transient ischemic attack (TIA) can then develop. Dissemination of infected embolic material into cerebral or meningeal vessels may lead to meningitis or brain abscesses. Cerebral hemorrhage is a rare neurological complication of IE. It is caused by a rupture of a mycotic aneurysm. It can occur months or years after the IE has been cured.

In patient with neurological manifestation, there could be symptoms and signs of multisystem embolisation. The frequency of major embolic events could be significantly higher in patients with vegetations on echocardiography. The size and nature of vegetation seems to be more important than presence of vegetation. According to some researchers, the size of vegetation more than 10 mm especially with native mitral valve infection, has significantly higher incidence of neurological events.

### Diagnosis

The main stay of diagnosis of infective endocarditis is blood culture and echocardiography. The most popular diagnostic criteria used is the Dukes criteria. The diagnosis of infective endocarditis based on these criteria are

a) Pathological critieria. Micro organisms demonstrated by culture or histology from lesions

b) Clinical criteria

Presence of two major criteria or one major criteria and three minor criteria or five minor criteria.

### Treatment

The treatment of IE includes

#### a) Initial stabilization

It would involve establishing and maintaining air way, breathing and circulation. In some cases endotracheal intubation could be needed. Valvular rupture can result in cardiac decompensation. Intra aortic balloon counter pulsation (IABP) might be necessary.

#### b) Empirical antibiotic therapy

On suspicion of IE, patient should be started on antibiotics. In a patient with uncomplicated history a popular regime is Ceftriaxone with Gentamicin.

#### c) Definitive antibiotic treatment

It should be based on culture and sensitivity results. In general antibiotics needs to be continued for 4 to 6 wks

#### d) Surgical treatment

Some indications for surgical treatment are, severe valvular dysfunction, such as acute congestive heart failure, major embolic complications, relapsing prosthetic valve IE, fungal IE, new conduction defects or arrhythmias after infection, or persistent bacteremia after appropriate antibiotic therapy.

There is a small role for extracardiac surgery in the treatment of neurological complication. Eg: Ruptured mycotic aneurysm.[9]

### Prognosis

The overall in-hospital mortality rate of IE is approximately 45%. In-hospital outcome of adult patients admitted to the ICU with infective endocarditis singularly bad. (11)Clinical factors in patients with native valve IE, associated with poor outcome are septic shock (odds ratio 4.81), cerebral emboli (3.00), immunocompromised state (2.88). Cardiac surgery has a positive influence on the mortality rate.(0.475), The patients who underwent cardiac surgery during the same hospitalization, had a better outcome than other patients.[10]Most complications occur early.

The mortality rates of IE is significantly more in patients with neurological manifestations. (2.4 times higher) at 3 months[10]. Patients with silent CVC (Cerebro Vascular Complication) or TIA have a relatively good prognosis, whereas those with stroke have significant excess mortality particularly in case of patients with mechanical prosthetic valve IE or impaired consciousness.

A simultaneous infection of the native aortic and mitral valves is associated with a poor prognosis. Mortality rate from this is about 56%. [9]

## Conclusion

The protean character of the IE, the latency of the cardiac symptoms and close simulation of other disorders combine to render the detection of IE peculiarly difficult. Clinicians should be aware of variety of cerebral presentations in patients with IE, since these may constitute the first symptoms of the disease which if untreated is always fatal.

Whenever a patient presents with agitation, if the cause is not clear, it is mandatory to go through all the causes of altered mental status however remote the chances of that may appear.

## Abbreviations

IV: Intra venous; GCS: Glasgow coma scale; IM: Intra muscular; CT: Compterised Tomography; ICU: Intensive care unit; IE: Infective endocarditis; NVE: Native valve endocarditis; PVE: Prosthetic valve endocarditis; PUO: Pyrexia of unknown origin; TIA: Transient ischemic attack; IABP: Intra aortic balloon pulsation.

## Consent

Written informed consent was obtained from the patient for publication of this case report. A copy of this written consent is available for review by the Editor – in – chief of this journal.

## Competing interests

The authors declare that they have no competing interests.

## Authors' contributions

KRM gathered data, analyzed data and played a key role in writing the case report. AM actively participated in literature review and writing the case report. JM supervised and guided us through the whole procedure whilst this was being written.
